# Texture Analysis of DCE-MRI Intratumoral Subregions to Identify Benign and Malignant Breast Tumors

**DOI:** 10.3389/fonc.2021.688182

**Published:** 2021-07-08

**Authors:** Bin Zhang, Lirong Song, Jiandong Yin

**Affiliations:** ^1^ School of Medicine and Bioinformatics Engineering, Northeastern University, Shenyang, China; ^2^ Department of Radiology, Shengjing Hospital of China Medical University, Shenyang, China

**Keywords:** breast tumors, magnetic resonance imaging, machine learning, texture analysis, DCE-MRI

## Abstract

**Purpose:**

To evaluate the potential of the texture features extracted from dynamic contrast-enhanced magnetic resonance imaging (DCE-MRI) intratumoral subregions to distinguish benign from malignant breast tumors.

**Materials and Methods:**

A total of 299 patients with pathologically verified breast tumors who underwent breast DCE-MRI examination were enrolled in this study, including 124 benign cases and 175 malignant cases. The whole tumor area was semi-automatically segmented on the basis of subtraction images of DCE-MRI in Matlab 2018b. According to the time to peak of the contrast agent, the whole tumor area was partitioned into three subregions: early, moderate, and late. A total of 467 texture features were extracted from the whole tumor area and the three subregions, respectively. Patients were divided into training (n = 209) and validation (n = 90) cohorts by different MRI scanners. The least absolute shrinkage and selection operator (LASSO) method was used to select the optimal feature subset in the training cohort. The Kolmogorov-Smirnov test was first performed on texture features selected by LASSO to test whether the samples followed a normal distribution. Two machine learning methods, decision tree (DT) and support vector machine (SVM), were used to establish classification models with a 10-fold cross-validation method. The performance of the classification models was evaluated with receiver operating characteristic (ROC) curves.

**Results:**

In the training cohort, the areas under the ROC curve (AUCs) for the DT_Whole model and SVM_Whole model were 0.744 and 0.806, respectively. In contrast, the AUCs of the DT_Early model (*P* = 0.004), DT_Late model (*P* = 0.015), SVM_Early model (*P* = 0.002), and SVM_Late model (*P* = 0.002) were significantly higher: 0.863 (95% CI, 0.808–0.906), 0.860 (95% CI, 0.806–0.904), 0.934 (95% CI, 0.891–0.963), and 0.921 (95% CI, 0.876–0.954), respectively. The SVM_Early model and SVM_Late model achieved better performance than the DT_Early model and DT_Late model (*P* = 0.003, 0.034, 0.008, and 0.026, respectively). In the validation cohort, the AUCs for the DT_Whole model and SVM_Whole model were 0.670 and 0.708, respectively. In comparison, the AUCs of the DT_Early model (*P* = 0.006), DT_Late model (*P* = 0.043), SVM_Early model (*P* = 0.001), and SVM_Late model (*P* = 0.007) were significantly higher: 0.839 (95% CI, 0.747–0.908), 0.784 (95% CI, 0.601–0.798), 0.890 (95% CI, 0.806–0.946), and 0.865 (95% CI, 0.777–0.928), respectively.

**Conclusion:**

The texture features from intratumoral subregions of breast DCE-MRI showed potential in identifying benign and malignant breast tumors.

## Introduction

Breast cancer is one of the most common cancers and the main cause of cancer deaths in women, accounting for approximately 30% of new cancer cases in women and 14% of cancer deaths (1). Advances in medical technology have resulted in a relatively high cure rate for early breast cancer through radiotherapy, chemotherapy, and surgery (2, 3). The treatment options for benign and malignant breast tumors differ, as do the local recurrence and survival rates (4). Benign breast tumors are generally curable through active treatment, whereas malignant tumors are difficult to cure and usually require surgery after neoadjuvant therapy to suppress local recurrence (5–7). Therefore, distinguishing benign from malignant breast tumors quickly and accurately is important.

Magnetic resonance imaging (MRI) is a non-invasive imaging method increasingly being used to detect and diagnose breast cancer. MRI has a higher sensitivity for the detection of breast lesions than mammography or breast ultrasound (8, 9). Among the available MRI methods, dynamic contrast-enhanced MRI (DCE-MRI) can provide tumor anatomical information and hemodynamic information with high spatial resolution, and it plays an important role in the diagnosis, differential diagnosis, and treatment response assessment of breast cancer (10–13). However, many benign lesions show strong contrast enhancement, which can lead to false-positive diagnoses, unnecessary biopsies, or overtreatment (14). The rate of preoperative breast DCE-MRI examinations is increasing, and an effective method for characterizing enhanced lesions is crucial to improve the accuracy of diagnosis.

Texture analysis refers to the extraction of texture feature parameters through specific image processing technology to obtain a quantitative or qualitative description of the texture (15, 16). Texture analysis is applied to breast MRI through image processing methods, which can be used to quantify the heterogeneity of lesions (17, 18). Studies have shown that texture features that characterize intratumoral heterogeneity can help identify benign and malignant breast tumors and distinguish molecular subtypes of breast cancer (19–21).

Previous studies have mainly extracted texture features from the whole tumor area in MRI images. However, the texture features derived from subregions within the breast tumor may provide valuable information to aid in clinical diagnosis and help patients develop personal treatment plans (22–25). Fan et al. (26) have shown that the texture features extracted from intratumoral subregions of DCE-MRI can be used to predict Ki-67 status in estrogen receptor (ER)-positive breast cancer. To our knowledge, no research has been performed on the identification of benign and malignant breast tumors on the basis of texture features extracted from intratumoral subregions of breast DCE-MRI. The purpose of this study was to evaluate the potential of the texture features extracted from DCE-MRI of intratumoral subregions for distinguishing benign and malignant breast tumors.

## Materials and Methods

### Study Cohort

**Figure 1:**
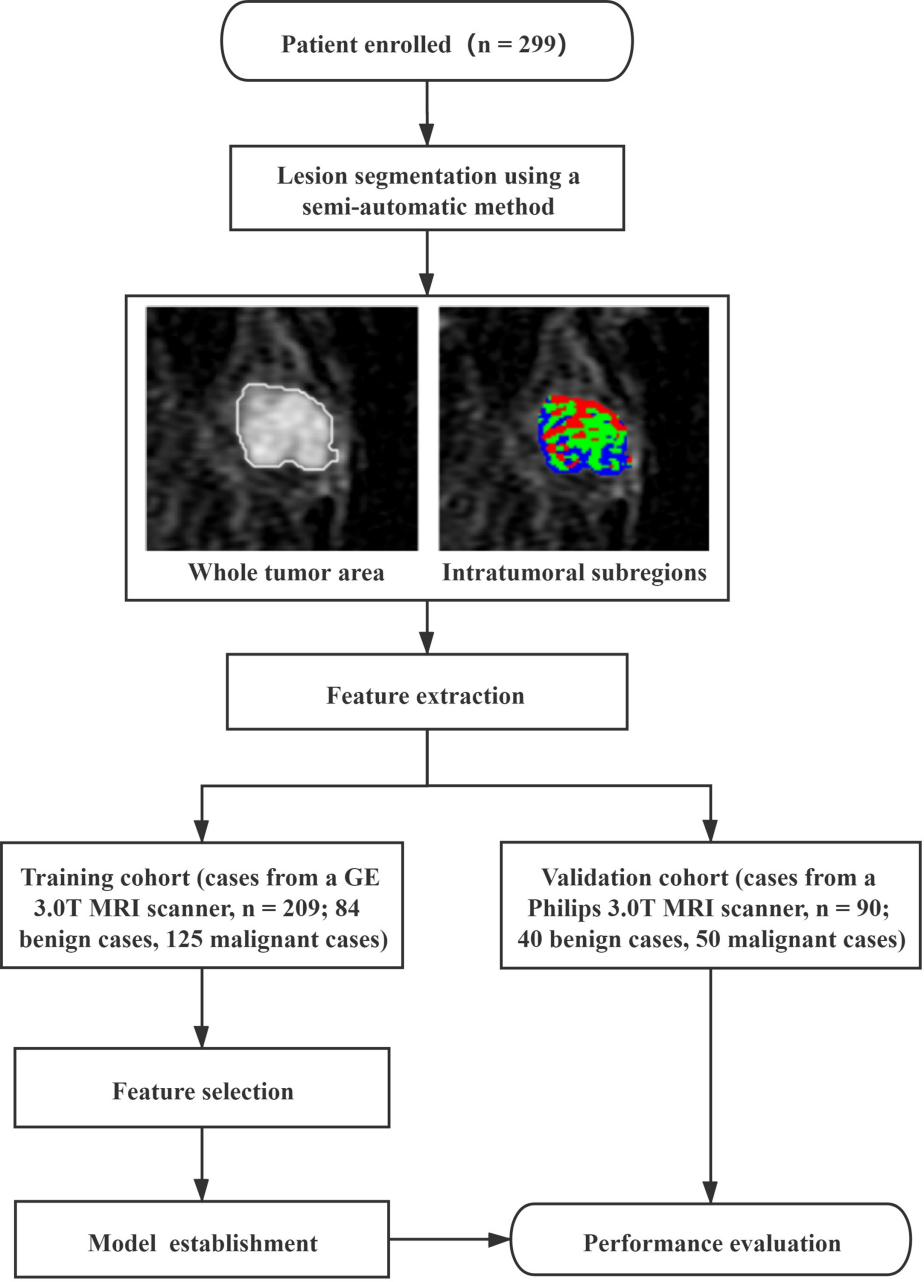
The flowchart adopted in this study.

This study was approved by the Ethics Review Committee at Shengjing Hospital of China Medical University (No. 2019PS175K), and the requirement for informed consent was waived because of the retrospective nature of the study. Between January 2017 and January 2020, patients who underwent breast DCE-MRI examinations were reviewed through the image archiving and communication system (PACS) at our institution. The study cohort initially included 378 patients. The inclusion criteria were as follows: (1) patients who underwent breast DCE-MRI and (2) patients with benign or malignant breast tumors confirmed by histopathology. The exclusion criteria were as follows: (1) patients treated with surgery, chemotherapy, or radiotherapy before DCE-MRI (n = 43); (2) patients diagnosed through excisional biopsy before DCE-MRI (n = 26); and (3) patients with insufficient image quality for subsequent processing because of obvious motion artifacts (n = 10). Consequently, 299 patients (mean age, 48.30 ± 9.74 years; range, 25–84 years) were divided into training (n = 209) and validation (n = 90) cohorts by different MRI scanners, including 124 benign and 175 malignant breast tumors. The clinical characteristics of the study cohort are summarized in **Table 1**. The flowchart of this study is shown in **Figure 1**.

**Table 1:** Clinical characteristics of the patients selected for this study.

Characteristic	Training cohort		Validation cohort	
	Number	%	Number	%
**Total patients**	209		90	
Benign (age range, 25–82 years)	84	40.2	40	44.4
Malignant (age range, 29–84 years)	125	59.8	50	55.6
**BI-RADS**				
3	18	8.6	7	7.8
4A	56	26.8	27	30
4B	43	20.6	16	17.8
4C	68	32.5	35	38.9
5	24	11.5	5	5.5
**Histopathological Type**				
Benign	84	40.2	40	44.4
Adenosis	48	23.0	23	25.5
Fibroadenoma	32	15.3	14	15.5
Papilloma	4	1.9	3	3.4
Malignant	125	59.8	50	55.6
Invasive carcinoma of no special type	116	55.5	41	45.6
Ductal carcinoma in situ	6	2.8	5	5.6
Invasive micropapillary carcinoma	2	1.0	3	3.3
Invasive lobular carcinoma	1	0.5	1	1.1

### Image Acquisition

In the training cohort, DCE-MRI examinations were performed with a GE 3.0T MRI scanner (Signa HDxt, GE Healthcare) equipped with a dedicated eight-channel bilateral breast coil on patients in a prone position. A transverse fat-suppression T1-weighted pre-contrast scan was first obtained with the VIBRANT-VX technique. Eight phases of fat-suppression T1-weighted post-contrast scans were acquired after intravenous injection of the contrast agent (Magnevist, Bayer-Schering Pharmaceuticals, Germany) at a dose of 0.15 mmol per kg body weight at 4 mlL/s and subsequent flushing with an equal volume of saline at the same injection speed. The following imaging parameters were used: repetition time (TR), 7.42 ms; echo time (TE), 4.25 ms; flip angle, 15°; slice thickness, 2.20 mm; spacing between slices, 2.20 mm; field of view, 340 × 340 mm^2^; image matrix, 1,024 × 1,024; slice number, 78. For each patient, eight phases of subtraction images were obtained by subtracting pre-contrast images from eight post-contrast images.

In the validation cohort, DCE-MRI examinations were performed with a Philips 3.0T MRI scanner (Ingenia, Philips Medical System, Best, Netherlands) equipped with a dedicated seven-channel bilateral breast coil with patient in a prone position. First, an axial fat-saturated T1-weighted pre-contrast scan was acquired. Then, eight axial contrast-enhanced fat-saturated T1-weighted scans were acquired after the intravenous bolus injection of the same contrast with the same dose. The imaging parameters were as follow: repetition time (TR), 4.14 ms; echo time (TE), 2.10 ms; flip angle, 12°; slice thickness, 2.00 mm; spacing between slices, 1.00 mm; field of view, 340 × 340 mm^2^; matrix, 380 × 380; slice number, 78. Eight subtraction sequences were obtained by subtracting the pre-contrast scan from each of the eight post-contrast scans.

### Image Processing and Lesion Segmentation

Two senior radiologists, with 10 and 15 years of experience in interpreting breast MRI were invited to review the subtraction images in the fourth phase and reached a consensus in selecting the slice image with the maximum tumor diameter for each patient for subsequent analysis (27). During the image review, the radiologists were blind to the patients’ pathological results. The whole tumor area was segmented with a semi-automatic method in Matlab 2018b (Mathworks, Natick, MA, USA), as described below (28, 29). One of the two radiologists manually delineated a region of interest (ROI) with an arbitrary shape around the lesion area on the subtraction image. The pixel gray levels within the ROI were first normalized to μ ± 3σ (μ: mean gray level of pixels within the ROI; σ: standard deviation), and the range was quantized to 8 bits/pixel to change the signal to noise ratio of the texture results (30–32). A spatial fuzzy C-means (FCM) algorithm was then used to delineate the contour boundary of the lesion according to the ROI, and the whole lesion area was refined through morphological processing methods (33–35). Another radiologist verified and proofread the results of the semi-automatic breast tumor segmentation.

### Intratumoral Subregion Partition

To better understand the intratumoral heterogeneity of breast tumors, as in a previous study (26), we divided the lesion area into three subregions according to the variations in pixel signal intensity in different phases. The specific partition details are as follows:

The relative enhancement of the post-contrast image compared with the pre-contrast image on a pixel-by-pixel basis was calculated with the following formula:

(1) $$H(m,n,t)=\frac{I(m,n,t)-I(m,n,t_{0})}{I(m,n,t_{0})}$$

where I(m, n, t) and I(m, n t_0_) represent the signal intensity of the pixel (m, n) captured at times t and t_0_ (the pre-contrast moment) (36). The time-signal intensity curve, H(m, n, t), was defined to describe the variation in the relative enhancement over time (37–39). The time to peak (TTP), which represents the arrival time of the peak relative enhancement, was calculated with the following formula:

(2) $$TTP(m,n)=\arg\underset{t}{\max}\;H(m,n,t)$$

Then the pixels within the tumor region were divided into three subregions according to their TTP values. More specifically, pixel sets at the first four, fifth or sixth, and seventh or eighth phases to achieve peak enhancement values were defined as early, moderate, and late subregions, respectively; this method was similar to those described in previous studies (26, 36). Therefore, the tumor was divided into three regions representing different sets of TTP values.

### Texture Feature Extraction

A total of 467 texture features were extracted from the whole tumor area and the three subregions with Matlab 2018b. The feature extraction methods could be classified into the following four categories: histogram, gray-level co-occurrence matrix (GLCM), gray-level run length matrix (GRLM), and discrete wavelet transform (DWT). Detailed information on the features is shown in **Table 2**. Each GLCM feature was calculated by using four angles (0, 45, 90, and 135°) and four distances (1, 2, 3, and 4 pixels). Each GRLM feature was calculated by using four angles (0, 45, 90, and 135°) and a distance of 1 pixel. In the following, (d, 0), (d, d), (0, d), and (-d, -d) were used to represent 0, 45, 90, and 135°, respectively, where d is the distance. Each DWT feature was calculated with four scales and three directions (horizontal, vertical, and diagonal) to generate low and high frequency components. In the following content, for example, Haar_2HH was used to represent the horizontal high frequency component of the second scale with the Haar wavelet.

**Table 2:** Detailed information on the extracted features.

Methods	Texture features	Number
Histogram	Mean, Kurtosis, Skewness, Variance	4
GLCM	Autocorrelation, Contrast, Correlation, Cluster prominence, Cluster shadow, Dissimilarity, Energy, Entropy, Homogeneity, Maximum probability, Sum of square, Sum average, Sum variance, Sum entropy, Difference square, Difference entropy, Information measure of correlation, Inverse difference normalized, Inverse difference moment normalized	380
GRLM	Short run emphasis, Long run emphasis, Gray-level non-uniformity, Run length non-uniformity, Fraction of image in runs, Low gray-level run emphasis, High gray-level run emphasis, Short run low gray-level emphasis, Short run high gray-level emphasis, Long run low gray-level emphasis, Long run high gray-level emphasis	44
DWT	Harr parameters	13
	Deubechies2 parameters	13
	Symlet4 parameters	13
Total		467

GLCM, gray-level co-occurrence matrix; GRLM, gray-level run length matrix; DWT, discrete wavelet transform.

### Feature Selection and Model Construction

To reduce the dimensionality of the features, the correlation between features was first tested with Pearson’s correlation analysis, and features with correlation coefficients of >0.95 relative to other features were removed. The remaining features were filtered by the least absolute shrinkage and selection operator (LASSO) method to select the optimal feature subset (40). Two machine learning models, decision tree (DT) and support vector machine (SVM), were used to construct classification models based on the optimal feature subset in the training cohort with a 10-fold cross-validation method for identifying benign and malignant breast tumors. And the classification models were tested by using a independent validation cohort. The 10-fold cross-validation refers to random division of the data set into 10 sets, nine of which were used for training and the last of which was used for testing. This process was repeated 10 times, and the test data differed each time.

### Statistical Analysis

All statistical analyses were performed in SPSS 22.0 (IBM, Armonk, NY, USA). The Kolmogorov-Smirnov test was first performed on texture features selected by LASSO to assess whether the samples followed a normal distribution (41); if so, the variables in the tables are represented by means ± standard deviation (SD), and if not, the variables in the tables are represented by medians ± interquartile range. Univariate logistic regression analysis was used to evaluate the performance of an independent feature in distinguishing benign from malignant breast tumors. The receiver operating characteristic (ROC) curve constructed in the professional statistics software MedCalc (version 14.10.20, http://www.medcalc.org/) was used to assess the classification performance by calculating the area under the ROC curve (AUC). The corresponding accuracy, sensitivity, and specificity were also determined. The DeLong test was used to determine the statistical significance of differences between AUCs. A two-tailed *P* value of <0.05 was considered statistically significant.

The intraobserver variability of texture features extracted by the two radiologists was evaluated by using intraclass correlation coefficients [ICC, (0, 0.4), poor agreement; (0.4, 0.6), moderate agreement; (0.6, 0.8), good agreement; and (0.8, 1), excellent agreement] (42, 43).

## Results

### Study Cohort

**Figure 2:**
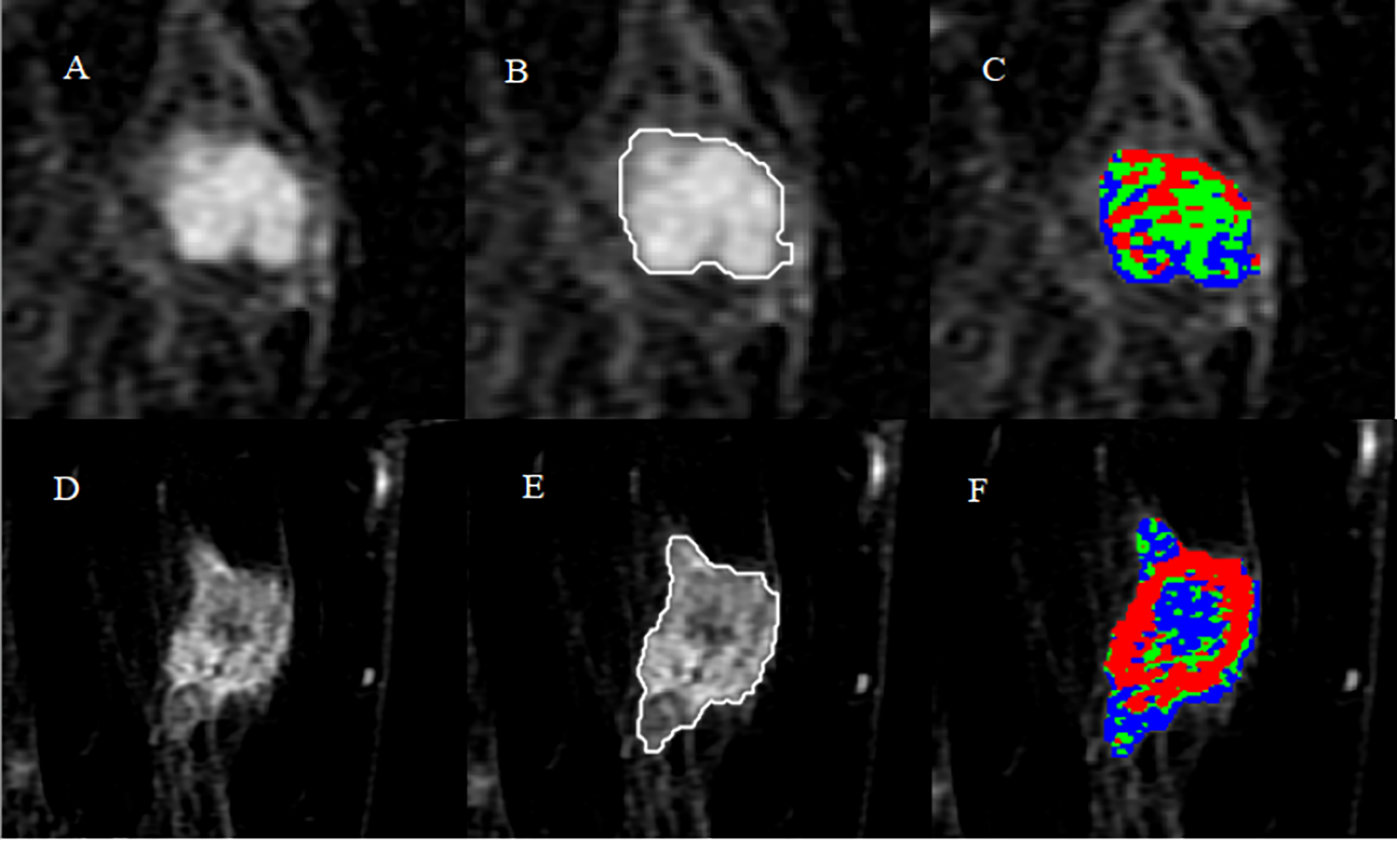
Results of whole tumor segmentation and intratumoral subregion partition. The first row shows the results of a benign case: **(A)** subtraction image with the maximum tumor diameter; **(B)** result of the whole tumor area segmented with a semi-automatic method; **(C)** result of intratumoral subregion partition, in which red, green, and blue represent the early, moderate, and late subregions, respectively. The second row shows the results of a malignant case: **(D)** subtraction image; **(E)** result of the whole tumor area; **(F)** result of intratumoral subregion partition.

A total of 299 patients were enrolled in this study. In the training cohort, the patients had 84 (40.2%) benign breast tumors classified into three histopathological types: adenosis (48), fibroadenoma (32), and papilloma (4). The 125 (59.8%) malignant breast tumors comprised 116 invasive carcinomas of no special type, 6 ductal carcinomas *in situ*, 2 invasive micropapillary carcinomas, and 1 invasive lobular carcinoma. In the validation cohort, the patients had 40 (44.4%) benign breast tumors classified into three histopathological types: adenosis (23), fibroadenoma (14), and papilloma (3). The 50 (55.6%) malignant breast tumors comprised 41 invasive carcinomas of no special type, 5 ductal carcinomas *in situ*, 3 invasive micropapillary carcinomas, and 1 invasive lobular carcinoma. The results of the whole tumor area segmentation and intratumoral subregion partition are displayed in **Figure 2**, which shows two randomly selected cases, one benign case and the other malignant case.

### Univariate Analysis

The results of univariate logistic regression analysis for identifying benign and malignant breast tumors are displayed in **Table 3**, which shows the top six features with the best performance extracted from the three subregions and the whole tumor area. The AUCs of features derived from the whole tumor area ranged from 0.732 to 0.786. Features from the early subregion performed best among the three subregions, with AUC values ranging from 0.787 to 0.886. The AUCs of the run length non-uniformity (1, 0) (*P* < 0.001), difference square (0, 1) (*P* = 0.004), and short run emphasis (1, 0) (*P* < 0.001) from the early subregion were significantly higher than those from the whole tumor area. The AUCs from the moderate subregion ranged from 0.715 to 0.777, and the AUCs from the late subregion ranged from 0.685 to 0.884. Among all individual features, the run length nonuniformity (1, 0) extracted from the early region achieved the highest AUC of 0.886 [95% confidence interval (CI), 0.836–0.926].

**Table 3:** Univariate analysis for predicting benign and malignant breast tumors.

Methods	Subregions	Features	AUC	95% CI	*P*-value^a^
Intratumoral subregions	Early	Run length nonuniformity (1, 0)	0.886	0.836–0.926	<0.001
		Difference square (0, 1)	0.877	0.825–0.918	0.004
		Short run emphasis (1, 0)	0.870	0.817–0.913	<0.001
		Correlation (−1, 0)	0.836	0.779–0.884	0.081
		Information measure of correlation (−2, 0)	0.820	0.761–0.870	0.391
		Deubechies2_2HH	0.787	0.725–0.840	0.186
	Moderate	Gray-level non-uniformity (1, 0)	0.777	0.715–0.832	<0.001
		Deubechies2_1VH	0.740	0.675–0.798	0.357
		Haar_1DH	0.736	0.671–0.795	<0.001
		Symlet4_1DH	0.729	0.664–0.788	0.016
		Deubechies2_1DH	0.718	0.651–0.778	0.003
		Mean	0.715	0.648–0.775	0.238
	Late	Information measure of correlation (0,1)	0.884	0.833–0.924	0.002
		Information measure of correlation (−1,0)	0.853	0.798–0.898	0.059
		Deubechies2_2VH	0.849	0.797–0.898	0.001
		Haar_1HH	0.840	0.784–0.887	0.001
		Haar_4HH	0.724	0.658–0.783	<0.001
		Mean	0.685	0.617–0.747	0.157
Whole tumor area	/	Deubechies2_2DH	0.786	0.725–0.840	/
		Haar_2DH	0.779	0.717–0.833	/
		Symlet4_2VH	0.776	0.713–0.831	/
		Symlet4_2HH	0.747	0.682–0.804	/
		Deubechies2_3DH	0.734	0.669–0.793	/
		Mean	0.732	0.667–0.791	/

AUC, area under the receiver operating characteristic curve; CI, confidence interval.

a P-value represents the comparison results of the features from the three intratumoral subregions and the same features from the whole tumor area.

The symbol ("/") represents null.

### Performance of Classification Models

**Figure 3:**
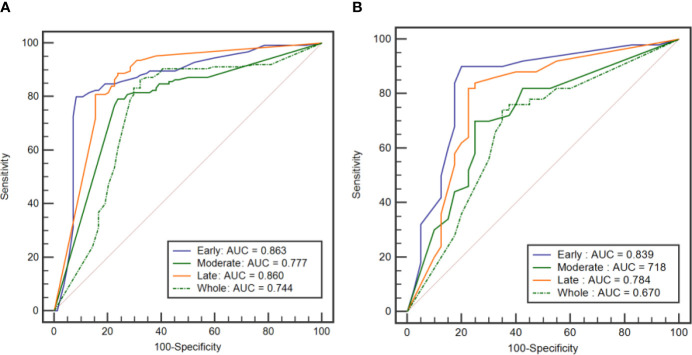
ROC curves of the DT classification models established by using the features extracted from the three intratumoral subregions and the whole tumor area. **(A)** ROC curves from the training cohort. **(B)** ROC curve from the external validation cohort.

**Figure 4:**
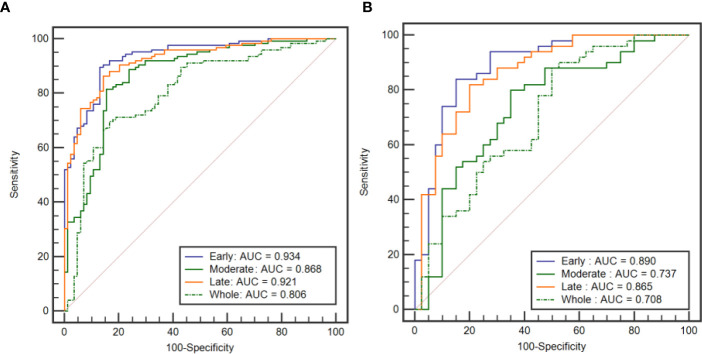
ROC curves of the SVM classification models established by using the features extracted from the three intratumoral subregions and the whole tumor area. **(A)** ROC curves from the training cohort. **(B)** ROC curves from the external validation cohort.

**Table 4** shows the performance of the classification models for distinguishing benign from malignant breast tumors in the training and validation cohorts, and the corresponding ROC curves are presented in **Figures 3** and **4**. In the training cohort, the AUCs of the DT_Whole model and SVM_Whole model were 0.744 and 0.806, respectively. In contrast, the AUCs of the DT_Early model (*P* = 0.004), DT_Late model (*P* = 0.015), SVM_Early model (*P* = 0.002), and SVM_Late model (*P* = 0.002) were significantly higher: 0.863 (95% CI, 0.808–0.906), 0.860 (95% CI, 0.806–0.904), 0.934 (95% CI, 0.891–0.963), and 0.921 (95% CI, 0.876–0.954), respectively. The SVM_Early model and SVM_Late model achieved better performance than the DT_Early model and DT_Late model (*P* = 0.003, 0.034, 0.008, and 0.026, respectively), as shown in **Table 5**. In the validation cohort, the AUCs of the DT_Whole model and SVM_Whole model were 0.670 and 0.708, respectively. In comparison, the AUCs of the DT_Early model (*P* = 0.006), DT_Late model (*P* = 0.043), SVM_Early model (*P* = 0.001), and SVM_Late model (*P* = 0.007) were significantly higher: 0.839 (95% CI, 0.747–0.908), 0.784 (95% CI, 0.601–0.798), 0.890 (95% CI, 0.806–0.946), and 0.865 (95% CI, 0.777–0.928), respectively. The SVM_Early model and SVM_Late model achieved better performance than the DT_Early model and DT_Late model (*P* = 0.018, 0.047, 0.035, and 0.029, respectively), as shown in **Table 6**. However, there was no significant difference between the SVM_Early model and the SVM_Late model in the training and validation cohorts (*P* = 0.524 and *P* = 0.523, respectively), and no significant difference between the DT_Early model and the DT_Late model (*P* = 0.945 and *P* = 0.332, respectively). Fifteen texture features extracted from the early subregion and 17 features extracted from the late subregion were selected by LASSO, as listed in **Table 7**.

**Table 4:** Performance of classification models for identifying benign and malignant breast tumors.

Models		Cohort	AUC	95% CI	Sensitivity	Specificity	Accuracy	*P*-value^a^
DT	Early	Training	0.863	0.808–0.906	80.0%	91.7%	79.8%	0.004
		Validation	0.839	0.747–0.908	90.0%	80.0%	77.8%	0.006
	Moderate	Training	0.777	0.715–0.832	79.2%	76.2%	76.5%	0.473
		Validation	0.718	0.613–0.808	70.0%	75.0%	74.4%	0.406
	Late	Training	0.860	0.806–0.904	80.8%	84.5%	78.5%	0.015
		Validation	0.784	0.601–0.798	82.0%	77.5%	76.7%	0.043
	Whole	Training	0.744	0.679–0.802	86.4%	67.9%	74.2%	/
		Validation	0.670	0.563–0.766	74.0%	65.0%	67.8%	/
SVM	Early	Training	0.934	0.891–0.963	89.6%	86.9%	88.5%	0.002
		Validation	0.890	0.806–0.946	84.0%	85.0%	83.3%	0.001
	Moderate	Training	0.868	0.814–0.911	81.6%	84.5%	80.4%	0.078
		Validation	0.737	0.634–0.824	80.0%	73.5%	72.2%	0.664
	Late	Training	0.921	0.876–0.954	86.4%	85.7%	84.5%	0.002
		Validation	0.865	0.777–0.928	82.0%	80.0%	80.0%	0.007
	Whole	Training	0.806	0.746–0.857	69.6%	83.3%	65.5%	/
		Validation	0.708	0.602–0.799	88.0%	67.5%	61.1%	/

AUC, area under the receiver operating characteristic curve; CI, confidence interval; DT, decision tree; SVM, support vector machine.

a P-value represents the comparison results of the AUC value of the same model established by features from intratumoral subregions and the whole tumor area..

The symbol ("/") represents null.

**Table 5:** *P*-values of DeLong tests between subregion models in the training cohort.

Classifier	DT_Early	DT_Moderate	DT_Late	SVM_Early	SVM_Moderate	SVM_Late
DT_Early	/	0.013	0.945	0.003	0.002	0.034
DT_Moderate	0.013	/	0.035	0.001	0.004	0.001
DT_Late	0.945	0.035	/	0.008	0.843	0.026
SVM_Early	0.003	0.001	0.008	/	0.020	0.524
SVM_Moderate	0.002	0.004	0.843	0.020	/	0.091
SVM_Late	0.034	0.001	0.026	0.524	0.091	/

DT, decision tree; SVM, support vector machine.

The symbol ("/") represents null.

**Table 6:** *P*-values of DeLong tests between subregion models in the validation cohort.

Classifier	DT_Early	DT_Moderate	DT_Late	SVM_Early	SVM_Moderate	SVM_Late
DT_Early	/	0.068	0.332	0.018	0.111	0.047
DT_Moderate	0.068	/	0.370	0.006	0.760	0.012
DT_Late	0.332	0.370	/	0.035	0.511	0.029
SVM_Early	0.018	0.006	0.035	/	0.007	0.523
SVM_Moderate	0.111	0.760	0.511	0.007	/	0.032
SVM_Late	0.047	0.012	0.029	0.523	0.032	/

The symbol ("/") represents null.

**Table 7:** Texture features extracted from early and late subregions selected with LASSO.

Features	Benign	Malignant
**Early subregion**		
Mean^a^	133.642 ± 41.162	168.686 ± 42.720
Variance^a^	27.638 ± 10.281	33.551 ± 8.434
Difference square (0, 1)^b^	0.220 ± 0.104	0.991 ± 0.357
Correlation (−2, 0)^b^	0.654 ± 0.259	0.823 ± 0.0987
Information measure of correlation (0, 1)^b^	0.6131 ± 0.186	0.822 ± 0.0691
Short run emphasis (1, 0)^b^	0.897 ± 0.0944	0.622 ± 0.114
Run length non-uniformity (1, 0)^b^	560.054 ± 13.620	426.963 ± 52.547
Deubechies2_2HH^b^	7.810 ± 2.364	4.399 ± 1.231
Deubechies2_1VH^b^	9.040 ± 4.241	4.853 ± 1.680
Symlet4_1VH^b^	8.174 ± 3.807	4.359 ± 1.445
Haar_4HH^b^	3.231 ± 1.749	5.317 ± 1.992
Deubechies2_3HH^b^	4.963 ± 1.313	3.826 ± 0.831
Symlet4_4VH^b^	3.089 ± 1.469	4.938 ± 1.845
Symlet4_1DH^b^	5.212 ± 2.273	2.517 ± 0.964
**Late subregion**		
Mean^a^	117.859 ± 29.076	136.495 ± 29.933
Variance^a^	30.496 ± 7.022	35.016 ± 7.631
Contrast (0,1)^b^	0.384 ± 0.203	0.716 ± 0.252
Information measure of correlation (0, 1)^a^	−0.595 ± 0.099	−0.441 ± 0.0807
Information measure of correlation (−1, 0)^b^	−0.594 ± 0.0807	−0.462 ± 0.0492
Short run emphasis (1, 0)^b^	0.672 ± 0.117	0.810 ± 0.149
Haar_1HH^a^	7.709 ± 4.446	13.814 ± 4.073
Deubechies2_2VH^a^	5.455 ± 2.949	9.708 ± 2.897
Haar_2HH^a^	6.651 ± 4.085	8.691 ± 2.746
Haar_4HH^b^	5.029 ± 1.993	2.895 ± 0.978
Haar_3VH^b^	4.496 ± 1.049	5.425 ± 1.343
Haar_4DH^b^	2.048 ± 0.881	1.567 ± 0.569
Deubechies2_3VH^b^	4.640 ± 1.481	5.489 ± 1.427
Deubechies2_4VH^b^	4.287 ± 1.630	3.103 ± 1.293
Deubechies2_4DH^b^	1.292 ± 0.393	1.835 ± 0.720
Symlet4_3HH^b^	3.335 ± 1.161	4.755 ± 1.129
Symlet4_3VH^b^	3.722 ± 1.434	5.611 ± 1.262

a The data are means ± SD.

b The data are medians ± interquartile range.

### Interobserver Agreement Evaluation

The texture features derived from the two groups of ROIs delineated independently by two radiologists showed excellent agreement [ICCs for whole lesion region, (0.875, 0.943); ICCs for early region, (0.853, 0.936); ICCs for moderate region, (0.837, 0.928); and ICCs for late region, (0.842, 0.931)].

## Discussion

This study investigated the relationship between texture features extracted from intratumoral subregions of breast DCE-MRI and the differential diagnosis of benign and malignant breast tumors. Features from subregions were able to distinguish benign from malignant breast tumors, and features from subregions representing the early and late TTP values achieved better performance than those from the whole tumor area in the training and validation cohorts. The SVM_Early model, SVM_Moderate model, and SVM_Late model demonstrated higher performance than the DT_Early model, DT_Moderate model, and DT_Late model, respectively.

Texture analysis can characterize intratumoral heterogeneity on the basis of quantitative image features extracted from conventional medical imaging to help diagnose, stage, and predict the prognosis and response to treatment in multiple oncology fields (44–46). Intratumoral heterogeneity reflects differences in biological characteristics, such as gene expression, metabolism, and angiogenesis (23, 47). Texture features derived from intratumoral subregions that reflect the heterogeneity of breast tumors, rather than the whole tumor area, may play a more important role in the prognostic analysis and identification of hormone receptor status in breast cancer (26, 36). A previous study has shown that texture features extracted from subregions with rapid delayed washout can be used to assess ER status and lymph node classification in breast cancer (48). Chang et al. (49) have quantified intratumoral heterogeneity on breast DCE-MRI by using a subregion-based feature extraction method for predicting ER status, human epidermal growth factor receptor 2 (HER2) status, and triple-negative breast cancer, achieving accuracy of 73.53, 82.35, and 77.45%, respectively. In this study, an intratumoral subregion partition method was used to distinguish benign from malignant breast tumors. Texture features were derived from three subregions and the whole tumor area, and the corresponding classification models were established. The models built with features from the early and late subregions achieved better performance than models built with features from the whole tumor area. A possible explanation for this finding is that the intratumoral subregions reflect angiogenesis, which may be indicative of the aggressiveness of malignant breast tumors (50).

A previous study has investigated the diagnostic performance of mammography texture analysis in differentiating benign from malignant breast tumors (51). In the present study, the subtraction images of DCE-MRI were used for texture analysis. Previous studies have discussed the roles of histograms, GLCM, and GRLM-based texture features in the differential diagnosis or treatment response assessment in breast cancer (14, 44). In addition to the features used in these studies, DWT-based features were extracted in this study. DWT is used to modify the image from the spatial domain to the frequency domain and has been extensively applied to feature extraction from electroencephalogram signals (52, 53). In the present univariate analysis, the DWT-based features derived from the late subregion, including Deubechies2_2VH (*P* = 0.001), Haar_1HH (*P* = 0.001), and Haar_4HH (*P* < 0.001), performed better in distinguishing benign from malignant breast tumors than those derived from the whole tumor area.

Two prevalent machine learning methods, DT and SVM, were applied to establish classification models in this study. To prevent overfitting, a 10-fold cross-validation method was used. The models established with features from the early and late subregions achieved better performance than models from the whole tumor area in the training and validation cohorts. However, no significant differences were found between the performance of models from the moderate subregion and that of models from the whole tumor area in the training and validation cohorts (*P* = 0.473 and *P* = 0.078, *P* = 0.406 and *P* = 0.664, respectively). Furthermore, the SVM_Early model, SVM_Moderate model, and SVM_Late model had higher AUCs than the DT_Early model, DT_Moderate model, and DT_Late model. SVM initially maps the input vector to a higher-dimensional feature space and identifies the hyperplane that divides the data points into two categories; the resulting classifier can reliably classify new samples and achieve considerable versatility (54).

A previous study by Li et al. (55) has applied four methods to classify benign and malignant breast tumors, and reported that the DT model achieved the best performance, with an AUC of 0.781, a sensitivity of 0.6, and a specificity of 0.894. Another study has used an SVM model for classifying benign and malignant breast tumors and obtained a sensitivity of 66.67% and a specificity of 93.55% (56). Wang et al. (20) have used logistic regression analysis to distinguish benign and malignant breast tumors, and achieved an accuracy of 79.5%, a sensitivity of 0.607, a specificity of 0.800, and an AUC of 0.802. In comparison, the best classification performance of our SVM_Early model achieved an AUC of 0.934, a sensitivity of 89.6%, a specificity of 86.9%, and an accuracy 88.5%. However, studies in which the classification model is based on deep learning methods have reported higher accuracy in distinguishing benign and malignant breast lesions (57, 58).

In addition, we separately evaluated the intraobserver variability of texture features extracted from the whole lesion region and from three different intratumoral subregions. The two radiologists showed high consistency in calculating texture features from the single-slice method, and all ICCs were greater than 0.8. The intraobserver variability was mainly related to slice selection and ROI delineation. Hence, standardized strategies for ROI determination are crucial.

This study has some limitations. First, the sample size was relatively small. Second, only a representative single-slice image was analyzed, and thus some useful information on the tumor might have been missed. Texture analysis based on three-dimensional breast tumor lesions may yield more useful information (59). Finally, the subtraction images of breast DCE-MRI were used to extract texture features. Features derived from post-contrast images or diffusion weighted imaging images may be helpful in distinguishing benign from malignant breast tumors (60).

## Conclusion

The texture features extracted from intratumoral subregions of breast DCE-MRI can be used as imaging biomarkers for the differential diagnosis of benign from malignant breast tumors. Specifically, features derived from subregions representing the early and late TTP values achieved better performance than features from the whole tumor area. Further research with a larger sample size is needed to verify the results of this study.

## Data Availability Statement

The original contributions presented in the study are included in the article/supplementary material. Further inquiries can be directed to the corresponding author.

## Ethics Statement

The studies involving human participants were reviewed and approved by Shengjing Hospital of China Medical University. Written informed consent for participation was not required for this study in accordance with the national legislation and the institutional requirements. Written informed consent was not obtained from the individual(s) for the publication of any potentially identifiable images or data included in this article.

## Author Contributions

Methodology, JY. Validation, LS. Investigation, BZ. Writing—original draft preparation, LS. Writing—review and editing, JY. Supervision, JY. Project administration, JY. Funding acquisition, JY. All authors contributed to the article and approved the submitted version.

## Funding

This research was supported by grants from the Research and Development (R&D) Foundation for Major Science and Technology from Shenyang (No. 19-112-4-105), the Big Data Foundation for Health Care from China Medical University (No. HMB201902105), the Natural Fund Guidance Plan from Liaoning (No. 2019-ZD-0743), and the 345 Talent Project from Shengjing Hospital of China Medical University.

## Conflict of Interest

The authors declare that the research was conducted in the absence of any commercial or financial relationships that could be construed as a potential conflict of interest.
